# The Matthews correlation coefficient (MCC) is more reliable than balanced accuracy, bookmaker informedness, and markedness in two-class confusion matrix evaluation

**DOI:** 10.1186/s13040-021-00244-z

**Published:** 2021-02-04

**Authors:** Davide Chicco, Niklas Tötsch, Giuseppe Jurman

**Affiliations:** 1 Krembil Research Institute, Toronto, Ontario, Canada; 2 Universität Duisburg-Essen, Essen, Germany; 3 Fondazione Bruno Kessler, Trento, Italy

**Keywords:** Matthews correlation coefficient, Balanced accuracy, Bookmaker informedness, Markedness, Confusion matrix, Binary classification, Machine learning

## Abstract

Evaluating binary classifications is a pivotal task in statistics and machine learning, because it can influence decisions in multiple areas, including for example prognosis or therapies of patients in critical conditions. The scientific community has not agreed on a general-purpose statistical indicator for evaluating two-class confusion matrices (having true positives, true negatives, false positives, and false negatives) yet, even if advantages of the Matthews correlation coefficient (MCC) over accuracy and F_1_ score have already been shown.In this manuscript, we reaffirm that MCC is a robust metric that summarizes the classifier performance in a single value, if positive and negative cases are of equal importance. We compare MCC to other metrics which value positive and negative cases equally: balanced accuracy (BA), bookmaker informedness (BM), and markedness (MK). We explain the mathematical relationships between MCC and these indicators, then show some use cases and a bioinformatics scenario where these metrics disagree and where MCC generates a more informative response.Additionally, we describe three exceptions where BM can be more appropriate: analyzing classifications where dataset prevalence is unrepresentative, comparing classifiers on different datasets, and assessing the random guessing level of a classifier. Except in these cases, we believe that MCC is the most informative among the single metrics discussed, and suggest it as standard measure for scientists of all fields. A Matthews correlation coefficient close to +1, in fact, means having high values for all the other confusion matrix metrics. The same cannot be said for balanced accuracy, markedness, bookmaker informedness, accuracy and F_1_ score.

**Supplementary Information:**

The online version contains supplementary material available at (10.1186/s13040-021-00244-z).

## Introduction

Evaluating the results of a binary classification remains an important challenge in machine learning and computational statistics. Every time researchers use an algorithm to discriminate the elements of a dataset having two conditions (for example, *positive* and *negative*), they can generate a contingency table called *two-class confusion matrix* representing how many elements were correctly predicted and how many were wrongly classified [1–8].

Among the positive data instances, the ones that the algorithm correctly identified as positive are called *true positives* (TP), while those wrongly classified as negative are labeled *false negatives* (FN). On the other side, the negative elements that are correctly labeled negative are called *true negatives* (TN), while those which are wrongly predicted as positives are called false positives (FP).

When the predicted values are real numbers ($\in \mathbb {R}$), one needs a cut-off threshold *τ* to discriminate between positives and negatives and properly fill the confusion matrix categories. The best practice suggests to compute the confusion matrices for all the possible cut-offs. Then, these confusion matrices can be used to generate a receiver operating characteristic (ROC) curve [9] or a precision-recall (PR) curve [10]. Finally, practitioners can compute the area under the curve (AUC) of the ROC curve or of the PR curve to evaluate the performance of the classification. The AUC ranges between 0 and 1: the closer to 1, the better the binary classification.

Although popular and useful, the PR curve and ROC curve can be employed only when the real prediction scores are available. Additionally, AUC as a metric suffers from several drawbacks, both when used for ROC curves [11, 12] and when used for PR curves [13]. The Precision-Recall curve, especially, has flaws similar to those of the F_1_ score, being based on the same statistical measures. To overcome these issues, Cao and colleagues [14] recently introduced a new curve based on MCC and F_1_ score.

If the predictive scores are binary (usually represented as zeros and ones), however, there is just a single confusion matrix to analyze. And to be informative, each category of the confusion matrix (TP, TN, FP, FN), must not be evaluated independently, but rather with respect to the other ones.

For this scope, scientists invented several confusion matrix rates in the past. Some of them involve only two confusion matrix categories: *sensitivity* (Eq. 1), *specificity* (Eq. 2), *precision* (Eq. 3), and *negative predictive value* (Eq. 4), among them, are particularly useful to recap the predictive quality of a confusion matrix. We refer to these four rates as *basic confusion matrix rates*.

1 $$ \text{true positive rate~(TPR)} = \frac{TP}{TP+FN}   $$

(worst value =0; best value =1)

2 $$ \text{true negative rate~(TNR)} = \frac{TN}{TN+FP}  $$

(worst value =0; best value =1)

3 $$ \text{positive predictive value~(PPV)} = \frac{TP}{TP+FP}  $$

(worst value =0; best value =1)

4 $$ \text{negative predictive value~(NPV)} = \frac{TN}{TN+FN}   $$

(worst value =0; best value =1)

True positive rate is also called *recall* or *sensitivity*. True negative rate is also known as *specificity*. Positive predictive value is also called *precision*.

These four binary rates indicate the ratios of correctly predicted positives (TP) with respect to the total number of positive data instances (sensitivity) and the total number of positive predictions (precision), and the ratios of correctly predicted negatives (TN) with respect to the total number of negative data instances (specificity) and the total number of negative predictions (negative predictive value).

Other confusion matrix scores involve three or even all the four confusion matrix categories, therefore providing a more complete and informative response: *Matthews correlation coefficient* (MCC) (Eq. 5), *accuracy*, *F*_1_ *score*, *balanced accuracy* (Eq. 6), *bookmaker informedness* (Eq. 7), and *markedness* (Eq. 8).

Matthews correlation coefficient (MCC), in fact, measures the correlation of the true classes *c* with the predicted labels *l*:

5 $$ \begin{array}{l} \text{MCC} \; = \frac{Cov(c, l)}{\sigma_{c} \cdot \sigma_{l}} = \frac{TP \cdot TN - FP \cdot FN}{\sqrt{(TP+FP)\cdot(TP+FN)\cdot(TN+FP)\cdot(TN+FN)}} \end{array}   $$

(worst value =−1; best value =+1) where *Cov*(*c*,*l*) is the covariance of the true classes *c* and predicted labels *l* whereas *σ*_*c*_ and *σ*_*l*_ are the standard deviations, respectively.

Balanced accuracy is the arithmetic mean of sensitivity and specificity (Eq. 6), and strongly relates to bookmaker informedness (Eq. 7). Markedness, instead, is the arithmetic mean of precision and negative predictive value (Eq. 8).

6 $$ \text{balanced accuracy~(BA)} = \frac{TPR+TNR}{2}   $$

(worst value =0; best value =1)

7 $$ \text{bookmaker informedness~(BM)} = {TPR+TNR-1}   $$

(worst value =−1; best value =+1)

8 $$ \text{markedness~(MK)} = {PPV+NPV-1}   $$

(worst value =−1; best value =+1)

Accuracy and F_1_ score, although popular among the scientific community, can be misleading [15, 16].

The Matthews correlation coefficient (Eq. 5) [17], instead, generates a high score only if the classifier correctly predicted most of the positive data instances and most of the negative data instances, and if most of its positive predictions and most of its negative predictions are correct.

Although Eq. 5 is undefined whenever the confusion matrix has a whole row or a whole column filled with zeros, by simple mathematical considerations it is possible to cover such cases and thus having MCC defined for all confusion matrices [15].

Unfortunately, this is not the case for BA, BM and MK, whose definition relies on the definition of the four basic rates TPR, TNR, PPV, NPV.

These four metrics have the shape $f(x,y)=\frac {x}{x+y}$, for *x*∈*TP*,*TN* and *y*∈*FP*,*FN*.

Now, $\underset {\underset {x=0,y>0}{(x,y)\to (0,0)}}{\lim }{f(x,y)=0}$, but $\underset {\underset {x=0,y>0}{(x,y)\to (0,0)}}{\lim }{f(x,y)=0}$ and $\underset {\underset {y=\frac {1-t}{t}x}{(x,y)\to (0,0)}}{\lim }{f(x,y)=0}$ for *t*∈(0,1).

This implies that ${{\lim }_{(x,y)\to (0,0)} \frac {x}{x+y}}$ does not exist, and thus there is no meaningful value these four metrics can be set to whenever their formula is not defined. Thus, when (*TP*+*FN*)·(*TN*+*FP*)=0 both BA and BM are undefined, and the same happens to MK when (*TP*+*FP*)·(*TN*+*FN*)=0.

Even if some studies forget about MCC [18, 19], it has been shown to be effective in multiple scientific tasks [20, 21].

Since the advantages of MCC over accuracy and F_1_ score have been already discussed in the scientific literature [15], in this paper we focus on the benefits of MCC over three other metrics: balanced accuracy (BA), bookmaker informedness (BM), and markedness (MK).

The scientific community has employed balanced accuracy for a long time, but its benefits over general accuracy were introduced by Brodersen and colleagues [22] in 2010, and reaffirmed by Wei et al. [3] a few years later. Several researchers employed balanced accuracy in different areas such as robotics [23], classical genetics [24–26], neuroimaging [27], computational biology [28], medical imaging [29, 30], molecular genetics [31], sports science [32], and computer vision applied to agriculture [33].

Also, Peterson and coauthors [34] showed that balanced accuracy can work well for feature selection, while García et al. [35] took advantage of it to build a new classification metric called *Index of Balanced Accuracy*.

Powers [36] introduced bookmaker informedness and markedness in 2003, but, to the best of our knowledge, these two measures have not become as popular as balanced accuracy in the machine learning community so far. Bookmaker informedness (BM) is identical to Peirce’s I and Youden’s index (also known as Youden’s J statistic) which have been introduced in 1884 and 1950, respectively [37, 38]. Youden’s index is often used to determine the optimal threshold *τ* for the confusion matrix [39–41].

Bookmaker informedness has been used in several studies, most authored by Powers [42–44], and few authored by other scientists (two in image recognition [45, 46], one in text mining [47], and one in vehicle tracking [48]). Two studies show the effectiveness of markedness in environmental sciences [49] and economics [50].

We organized the rest of the paper as follows. After this Introduction, we describe the mathematical foundations of the analyzed rates in terms of confusion matrix (“Mathematical background” section). We then report and discuss our discoveries regarding the relationships between MCC and balanced accuracy, bookmaker informedness, and markedness, describing some use cases and a real bioinformatics scenario (“Results and discussion” section). We conclude this study by drawing some conclusions about our analyses and describing some potential future development (“Conclusions” section).

## Mathematical background

As mentioned in the introduction, the entries of the confusion matrix (TP, TN, FP, FN) are not meaningful individually but rather when they are interpreted relative to each other. In this section, we introduce a redefinition with individually meaningful dimensions which also proves helpful in the comparison of metrics.

### Prevalence and bias

Prevalence (*ϕ*) measures how likely positive cases are in the test set

9 $$ \phi = \frac{TP + FN}{N}  $$

where N represents the sample size of the test set. *ϕ* is independent of the classifier. On the other hand, bias (*β*) measures how likely the classifier is to predict positive for the test set.

10 $$ \beta = \frac{TP + FP}{N} = TPR \cdot \phi + (1 - TNR) \cdot (1- \phi)  $$

*β* is dependent both on the underlying test set as well as the classifier because true positive rate (TPR) and true negative rate (TNR) are classifier intrinsic.

We note that *ϕ* in a test dataset is often arbitrary. In many medical studies, for example, the *ϕ* in the test dataset is completely unrelated to the prevalence in the population of interest. This is due to the fact that the dataset was provided by physicians treating patients suffering from a disease. Consequently, they have data on a limited number of sick patients and a cohort of controls. Depending on the size of the control group, the prevalence will vary. Unless the control group size is chosen in a way that the prevalence in the overall dataset equals the prevalence in the population of interest, that means new subjects being tested for the disease, the prevalence of the dataset is arbitrary.

Precision and negative predictive value depend on *ϕ* (“Interpretation of high values of multi-category metrics” subsection). Since MK and MCC depend on precision and negative predictive value, they are also affected by *ϕ*. If the prevalence in the dataset is arbitrary and not reflective of the prevalence in the population of interest, these metrics will also not reflect the performance of the classifier for the population of interest. In this case, it might be best to ignore these metrics because they are not meaningful for the application to the population of interest.

### Redefining confusion matrix in terms of prevalence, TPR and TNR

All entries of a confusion matrix can be derived if N, *ϕ*, TPR and TNR are known [51, 52]. They can be represented in this way:

11 $$\begin{array}{*{20}l} TP &= N \cdot TPR \cdot {\phi} \end{array} $$

12 $$\begin{array}{*{20}l} FN &= N \cdot (1 - TPR) \cdot {\phi} \end{array} $$

13 $$\begin{array}{*{20}l} TN &= N \cdot TNR \cdot (1- {\phi}) \end{array} $$

14 $$\begin{array}{*{20}l} FP &= N \cdot (1 - TNR) \cdot (1- {\phi}) \end{array} $$

N and *ϕ* are entirely intrinsic to the dataset, and if N is small, there are only few observations. As in all scientific studies, small sample sizes limit the reliability of the conclusions that can be drawn from the data. Since metrics such as MCC, BM and MK are calculated from the confusion matrices, a small N leads to uncertainty in those metrics [53]. In practical applications, one needs to determine this uncertainty as described in the literature [22, 53].

In this manuscript, we define examples of confusion matrices to illustrate similarities and discrepancies between different metrics. Since MCC, BM and MK are independent of N, we decided to compare the metrics for relative confusion matrices, that contain the shares of the four categories (TP, TN, FP, and FN) rather than the counts, whose sum equals 1. This way, N, and hence uncertainty, become irrelevant.

However, we advise against using relative confusion matrices to evaluate real-world applications. It is important to communicate N in order to estimate how reliable the obtained results are. For each use case, we display examples of corresponding count confusion matrices alongside relative confusion matrices for the readership’s convenience.

TPR and TNR describe the classifier completely. Therefore, any metric that depends on *ϕ* (such as PPV, NPV, MCC) does not describe the classifier objectively but rather the performance of the classifier for a given dataset or prevalence. As mentioned earlier (“Prevalence and bias” section), *ϕ* in the test set is not necessarily representative of the *ϕ* in the population of interest. Therefore, one has to keep in mind if any metric including *ϕ* of the test set is of real interest.

This redefinition also sheds light onto a shortcoming that MCC, BM, BA, and MK have in common. All of these metrics try to capture the performance of a classifier in a single metric, whereas actually there are three relevant ones (prevalence, TPR, TNR). Reducing three dimensions into one leads to a loss of information. None of MCC, BM and markedness (MK) can be as informative individually as the complete confusion matrix (CM). A single metric is easier to interpret, though, and can under some conditions summarize the quality of the classifier sufficiently well.

## Results and discussion

Our results can be categorized in four parts. First, we demonstrate that balanced accuracy (BA) and BM are tightly related and can be used interchangeably (“BA and BM contain equivalent information” subsection). Second, we derive the relationships between MCC, BA, BM and MK (“Relationship between MCC and BA, BM, MK” subsection). Third, we elucidate how strongly the metrics can deviate for the same confusion matrix by showcasing selected use cases in greater detail (“Disagreements between MCC and BM” subsection). Finally, we investigate which metric can truthfully measure how similar a classifier behaves to random guessing (“Bookmaker informedness is the only metric that measures how similar a classifier is to random guessing” subsection).

### BA and BM contain equivalent information

BA and BM were developed independently by Brodersen and Powers [22, 54]. Inserting Eqs. 6 into 7 yields:

15 $$ BA = \frac{BM + 1}{2}   $$

Ultimately, BA and BM contain the same information. Both describe the classifier independently of the test dataset. The accessible range is the only difference: BA ranges from 0 to 1 whereas BM ranges from –1 to 1. All conclusions drawn for BM can be transferred to BA. In the rest of the manuscript, we will focus on the comparison between BM and MCC because the ranges correspond.

### Relationship between MCC and BA, BM, MK

To understand the differences between the metrics, we need to derive their mathematical relationships.

**Relationship between MCC and BM**. MCC can be expressed in multiple ways. An alternative to Eq. 5 is:

16 $$ \begin{array}{l} MCC = \sqrt{PPV \cdot TPR \cdot TNR \cdot NPV} - \sqrt{FDR \cdot FNR \cdot FPR \cdot FOR} \end{array}   $$

We report the definition of FDR, FNR, FPR, FOR in the Supplementary information. Based on the redefinition of the entries of the confusion matrix given earlier (“Redefining confusion matrix in terms of prevalence, TPR and TNR” subsection), one can also express all metrics in terms of N, *ϕ*, TPR and TNR. This approach facilitates deriving their relationships and leads to:

17 $$\begin{array}{*{20}l} & {MCC}= \end{array} $$

18 $$\begin{array}{*{20}l} &= { \sqrt{TPR \cdot \frac{{\phi}}{{\beta}} \cdot TPR \cdot TNR \cdot TNR \cdot \frac{1 - {\phi}}{1 - {\beta}}}}  + \\ & { - \sqrt{(1 - TNR) \cdot \frac{1 - {\phi}}{{\beta}} \cdot (1 - TPR) \cdot (1 - TNR) \cdot (1 - TPR) \cdot \frac{{\phi}}{1 - {\beta}}} =} \end{array} $$

19 $$\begin{array}{*{20}l} &= \sqrt{\frac{{\phi}} {{\beta}} \cdot \frac{1 - {\phi}}{1 - {\beta}}} \cdot \left[ TPR \cdot TNR - (1 - TPR) \cdot (1 - TNR) \right] = \end{array} $$

20 $$\begin{array}{*{20}l} &= \sqrt{\frac{{\phi} - {\phi}^{2}}{{\beta} - {\beta}^{2}}} \cdot (TPR + TNR - 1) = \end{array} $$

21 $$\begin{array}{*{20}l} &= \sqrt{\frac{{\phi} - {\phi}^{2}}{{\beta} - {\beta}^{2}}} \cdot {BM}  \end{array} $$

Equation 21 shows us that MCC and BM always yield the same sign, that means that both inform us if a classifier is informative or deceptive. While BM exclusively describes classifier intrinsic TPR and TNR, MCC is also dependent on data intrinsic *ϕ* as well as *β* which depends on both the classifier and the dataset.

Based on the $\sqrt {\frac {{\phi } - {\phi }^{2}}{{\beta } - {\beta }^{2}}}$ factor, MCC can be larger or smaller than BM. The terms *ϕ*−*ϕ*^2^ and *β*−*β*^2^ are symmetric around 0.5 and peak at this value. If the imbalance in the dataset (measured by |0.5−*ϕ*|) is larger than the imbalance in the predictions (measured by |0.5−*β*|), MCC is lower than BM and *vice versa*.

Moreover, Eq. 21 shows that rankings of two classifiers *A* and *B* based on MCC and BM can differ:

22 $$\begin{array}{*{20}l} MCC_{A} &> MCC_{B}  \end{array} $$

23 $$\begin{array}{*{20}l} BM_{B} &> BM_{A} \end{array} $$

Substituting Eq. 21 into Inequation 22 leads to:

24 $$ BM_{B} > BM_{A} > \sqrt{\frac{{\beta}_{A} - {\beta}_{A}^{2}}{{\beta}_{B} - {\beta}_{B}^{2}}} \cdot BM_{B}   $$

Since Inequation 24 can be fulfilled, using BM or MCC might lead to different conclusions on which classifier performs best. We show an example ranking different classifiers in a bioinformatics scenario in “Ranking of classifiers in a real bioinformatics scenario” subsection.

**Relationship between MCC and MK**. MK is defined similarly to BM but based on positive predictive value (PPV) and negative predictive value (NPV) instead of TPR and TNR:

25 $$ MK = PPV + NPV - 1  $$

Analogously to Eq. 21 we arrive at:

26 $$ {MCC} = \sqrt{\frac{{\beta} - {\beta}^{2}}{{\phi} - {\phi}^{2}}} \cdot {MK}   $$

MCC is larger than MK if the imbalance in the dataset is larger than the imbalance in the predictions, and *vice versa*. Equation 26 leads us to:

27 $$ MK = \frac{{\phi} - {\phi}^{2}}{{\beta} - {\beta}^{2}} \cdot {BM}  $$

MK is even more impacted by *ϕ* and *β* than MCC. Its results are strongly biased by class and prediction imbalance. Substituting Eq. 26 into Eq. 21 leads to:

28 $$ MCC = \pm \sqrt{{MK} \cdot {BM}}  $$

MCC is the geometric mean of BM and MK, as has been previously shown by Powers [54]. Interpreting MCC this way, it becomes obvious that MCC is only high if the classifier is well informed (BM is high) and if the real class is marked by the predicted label (MK is high).

**Fig. 1:**
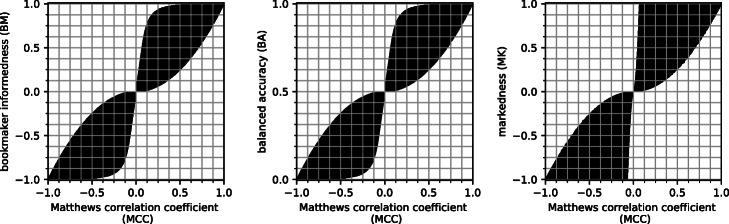
Relationships between MCC and BM, BA, and MK. Plots indicating the values of MCC in relationship with BM (left), BA (centre), and MK (right) calculated for approximately 8 million confusion matrices with 40 thousand samples each

**Visual comparison and correlation between MCC and BA, BM, MK**. To better understand the correlation between MCC and BA, between MCC and BM, and between MCC and MK, we depicted three scatterplots having the Matthews correlation coefficient on the *x* axis and each of the three other rates on the *y* axis (Fig. 1). We take advantage of these scatterplots to overview the mutual relations between MCC and the other rates.

As one can notice, the shape of the scatterplot of MCC and BM (Fig. 1, left plot) is identical to the one of MCC and BA (Fig. 1, central plot), only the scales on the *y* axis differ. BM, in fact, ranges from –1 to +1, while balanced accuracy’s possible values go from 0 to 1.

For each pair, the two measures are reasonably concordant, and close to the *x*=*y* straight line. However, the scatterplot clouds are wide, especially when MCC ranges between +0.1 and +0.9 and between –0.9 and –0.1, implying that for each value of MCC there are several values of BM, BA, and especially MK (and viceversa).

Overall, these scatterplots show that MCC is concordant with BM, BA, and MK when the analyzed confusion matrices generate high true positive rate, high true negative rate, high positive predictive value, and high negative predictive value.

However, the three plots also show some contradictions between pairs of rates when a classifier performs well just for one of the two binary classes. For example, when MCC is +0.2 and indicates poor performance, all the three other indicators can reach 0.9, meaning almost perfect predictions. We will discuss these contradictory messages later in the use cases UC1, UC2, and UC3 of the next section.

We conclude this section by quantitatively assessing the linear relation among the metrics in terms of the Pearson correlation coefficient (PCC) [55], a measure of linearity between two sets of values. Interpreting the two sets of values as coordinates of points, PCC is +1 when the points lie on a line with positive slope, PCC is –1 if the line has negative slope, PCC is zero if the points are spread out on the plane.

**Fig. 2:**
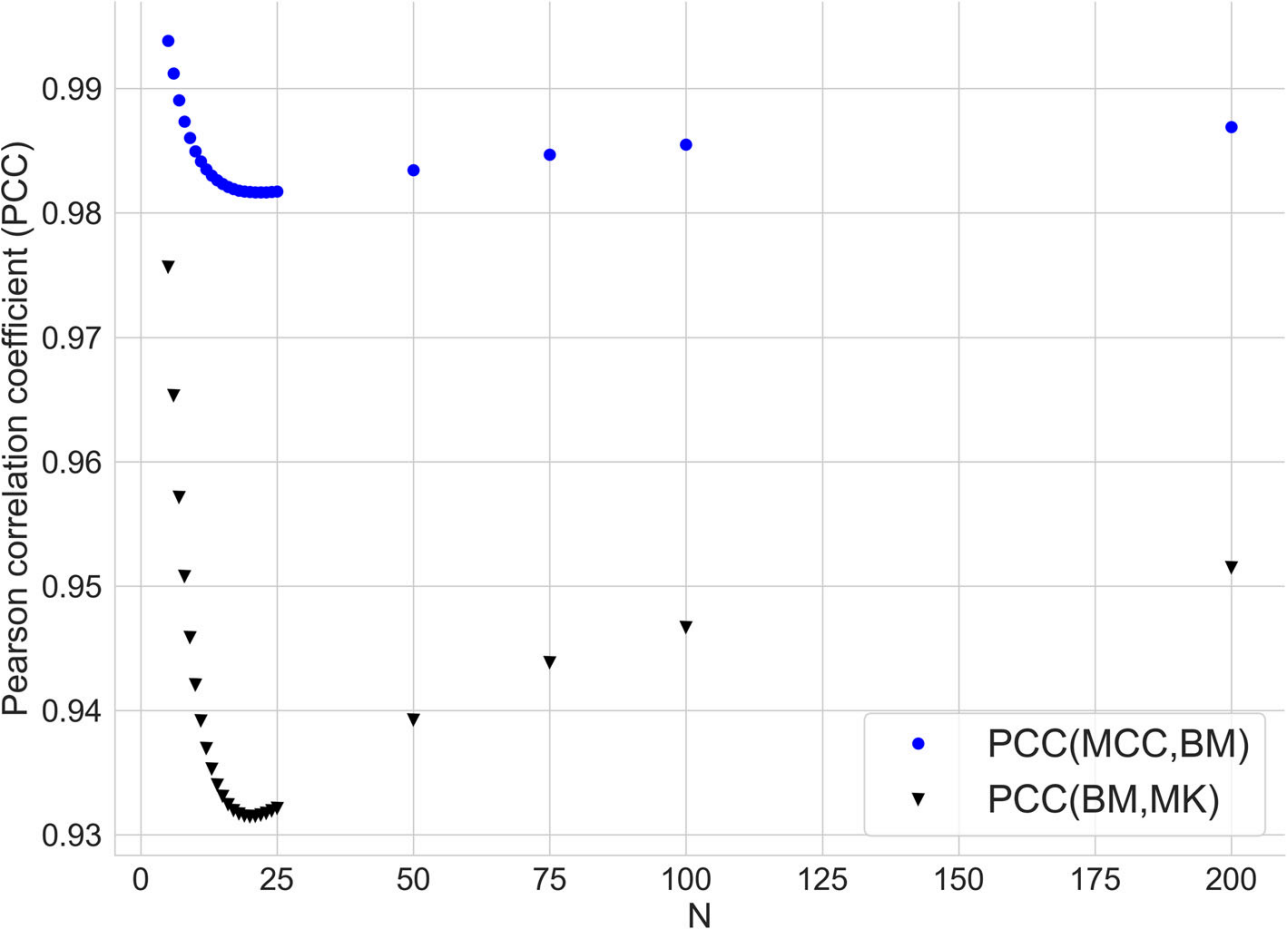
Pearson correlation between MCC, BM and MK as a function of number of samples *N*

For a given positive integer *N* and for a dataset with *N* samples, we consider all the possible $\binom {N+3}{3}$ confusion matrices and, for each matrix, we compute the corresponding MCC, BM and MK and then the Pearson correlation coefficient between MCC and BM, MCC and MK and BM and MK, respectively. We list the resulting PCC values in the Supplementary information, and plot them in Fig. 2.

Note that, since BA and BM are analytically linked by the affine map (Eq. 15), their mutual Pearson correlation coefficient is one, and thus we only consider BM in the current analysis. All three series of PCC values are very high (close to 1), denoting a strong linear relation between the three metrics. In details, PCC is slightly decreasing reaching the minimum around *N*≈25, then it increases, first quickly and then very slowly. Further, PCC(MCC, BM) coincides with PCC(MCC, MK) for each *N*, even if BM and MK do not coincide: this aspect comes with no surprise, due to the symmetric form of their expressions (Eqs. 7 and 8).

### Disagreements between MCC and BM

While *MCC*≈*BM*≈*MK* in many instances, the most insightful cases are those where the three metrics generate different results (Fig. 1). We present several use cases to illustrate why outcomes may differ depending on the metric and how to interpret them.

#### High TPR and TNR, very low prevalence

**Use case UC1**. In this example we study a classification experiment with high TPR and TNR, 0.99 and 0.95 respectively, and a very imbalanced dataset. Let us consider the following relative confusion matrix:

29 $$ relative \; {CM}1 = \left[\begin{array}{cc} {TP} = 9.99 \times 10^{-4} & {FN} = 9.99 \times 10^{-6} \\ {FP} = 0.05 & {TN} = 0.95 \end{array}\right]   $$

Assuming a sample size of 100,001, CM1 is an exemplary confusion matrix:

30 $$ {CM}1 = \left[\begin{array}{cc} {TP} = 100 & {FN} = 1 \\ {FP} = 5\;000 & {TN} = 94\;900 \end{array}\right]   $$

MCC is 0.136, indicating that predictions from the classifier do not correlate well with the real class. One should not make the mistake and consider the classifier to be similar to random guessing (which always has BM=MCC=0). Objectively, the classifier is well informed (BM = 0.94). Nevertheless, it clearly struggles to predict well for the given very small *ϕ*.

Ultimately, MCC and BM answer different questions. BM informs us that the classifier is substantially better than random guessing. Random guessing has BM = 0.0 because it produces true positive (TP) at the same rate of false positive (FP). In UC1, this is clearly not the case. The classifier predicts approximately 99% of reference positives as positive and 95% of all reference negatives as negative. UC1 demonstrates that MCC in general *does not* tell us if a classifier performs similarly to random guessing because it is biased by class imbalance [52]. We are going to discuss this in greater detail later (“Bookmaker informedness is the only metric that measures how similar a classifier is to random guessing” subsection). In fact, the low MCC value warns us that correlation between predicted and true class is low. This is equivalent to at least one of PPV, TPR, TNR, NPV being low (Eq. 16), in this case PPV ≈0.02.

A practical question would be if the classifier at hand is useful for practitioners, for example to predict a disease. The results in Table 1 show us that applying the test considerably increases our knowledge about the probability that a patient is infected. A positive test result raises the probability to be infected by a factor of 19. Nevertheless, even those patients that test positive are unlikely to be sick. On the other hand, those that receive a negative test result are extremely unlikely to be sick. These findings illustrate that positive test results should not be taken at face value. Its discriminative power is not sufficient for the tremendous class imbalance in the dataset. If this is required, a classifier with an even higher TPR and TNR needs to be built in order to increase PPV. Yet, the present classifier might still be helpful to identify patients which are almost certainly healthy (receiving negative test results) from those that could profit from additional diagnostic tests (receiving positive test results).

**Table 1:** Probability for positive data instance in UC1

Before testing	*ϕ*	0.001
Testing positive	PPV	0.019
Testing negative	1 – NPV	0.00001

The probability for a positive data instance, for example a patient that is truly sick, depends on the test result. While a positive test result increases the probability substantially, it remains low. A negative test result decreases it

#### Misleading BA and BM, informative MCC and MK

**Use case UC2**. Here we analyze a use case where we have a very high number of true positives, a high number of false negatives, and a very low number of false positives and true negatives (Eq. 31):

31 $$ relative \; {CM}2 = \left[\begin{array}{ll} {TP} = 8.99 \times 10^{-1}&{FN} = 9.99 \times 10^{-2} \\ {FP} = 9.99 \times 10^{-6} &{TN} = 8.99 \times 10^{-5} \end{array}\right]   $$

If the sample size is 100,010, CM2 is an exemplary confusion matrix:

32 $$ {CM}2 = \left[\begin{array}{cc} {TP} = 90,000 &{FN} = 10,000 \\ {FP} = 1 &{TN} = 9 \end{array}\right]   $$

We see that the classifier generates a large number of FN with respect to the number of TN. Therefore, we can intuitively state that this classifier performed poorly: its negative predictions are unreliable, since the NPV equals to 0.001.

Computing the confusion matrix rates, we can notice that the scores generated by the Matthews correlation coefficient (MCC = +0.027) and the markedness (MK = 0.001) confirm this messages: values around zero, in fact, mean inefficient classification.

The values of balanced accuracy and bookmaker informedness, however, contradict MCC and MK. For this confusion matrix, in fact, we have BA = 0.9 and BM = 0.8, which mean *almost perfect prediction*. This is a use case where BA and BM are clearly misleading: they do not represent the low ratio of true negatives (TN= 8.99×10^−5^) over false negatives (FN= 9.99×10^−2^), that means the low negative predictive value, in this confusion matrix (Eq. 31).

If a practitioner decided to evaluate this UC2 confusion matrix by only analyzing its corresponding balanced accuracy and bookmaker informedness, she/he would overoptimistically think that the classifier generated excellent predictions. The analysis of the results achieved by MCC and MK, instead, would have kept her/him on the right track.

This conclusion closely resembles the one from UC1, because Eq. 31 would be similar to Eq. 29 if class labels were swapped. Unlike F_1_ score, all the rates analyzed in this study (MCC, BA, BM, and MK) have the advantage to be invariant to class label swapping.

#### Virtually uninformed classifier, slightly elevated MCC, high MK

**Use case UC3**. Whereas we have discussed examples of low MCC opposed to high BM in UC1 and UC2, this use case UC3 will elaborate on the interpretation of low BM and moderate MCC. Let us consider the following confusion matrix CM3 (Eq. 33):

33 $$ relative \; {CM}3 = \left[\begin{array}{cc} {TP} = 0.999877792 & {FN} = 0 \\ {FP} = 1.11 \times 10^{-4} & {TN} = 1.11 \times 10^{-5} \end{array}\right]   $$

If the sample size is 90,011, CM3 is an example of confusion matrix:

34 $$ {CM}3 = \left[\begin{array}{cc} {TP} =90\;000 & {FN} = 0 \\ {FP} = 10 & {TN} = 1 \end{array}\right]   $$

Based on Eq. 33 one finds BM=0.091. The classifier at hand is only marginally better than a “silly” rule predicting positive in all cases, which would lead to BM = 0. Both have TPR = 1. Always predicting a positive class would lead to TNR = 0, while the presented classifier has TNR = 0.091.

The MCC of CM3 is +0.301. While one would not consider the classifier to be good based on this MCC, one would have a more favorable impression than based on BM. MK is close to 1, that means almost perfect, because PPV is approximately 1 and NPV is exactly 1. Since MCC is the geometric mean of BM and MK and MK is approximately 1, $MCC \approx \sqrt {BM}$. This demonstrates that medium values of MCC cannot be interpreted in the same way as medium values of BM.

Also, the correlation between predictions and ground truth is higher than the informedness. Predictions in UC3, as measured by MK, are very reliable, not because of a well informed classifier but rather because of the high *ϕ*.

This use case proves that MK does not reliably tell us how similar a classifier is to random predictions. On the contrary, while the classifier is poorly informed, MK is close to its maximum.

#### BM allows comparison across datasets, MCC does not

**Use case UC4**. The fact that MCC is dependent on *ϕ* leads to non-transferability across datasets. This aspect needs to be considered when searching the scientific literature for published classifiers that predict the same condition, for example a given disease.

Consider the following example. There are two publications A and B, describing two different classifiers, for example neural networks with different architectures [56], listing the corresponding confusion matrices and metrics (Table 2). You assume that the underlying datasets contain comparable samples. Based on the publications, one would like to decide which classifier/architecture is preferable.

**Table 2:** Evaluation of two classifiers A and B on separate datasets

Classifier	dataset	TP	FN	TN	FP	*ϕ*	TPR	TNR	BM	MCC		
(a) Relative CM												
A	1	0.35	0.15	0.35	0.15	0.5	0.7	0.7	0.4	0.4		
B	2	0.04	0.01	0.76	0.19	0.05	0.8	0.8	0.6	0.3		
Classifier	dataset	TP	FN	TN	FP	*ϕ*	TPR	TNR	BM	MCC	n+	n–
(b) Exemplary CM for a sample size of 200												
A	1	70	30	70	30	0.5	0.7	0.7	0.4	0.4	100	100
B	2	8	2	152	38	0.05	0.8	0.8	0.6	0.3	10	190

In the literature, different publications compare classifiers for the same task on separate datasets. This poses a problem for the comparability of metrics which are dependent on prevalence

Comparing the Matthews correlation coefficients (Table 2), one would opt for classifier A. Objectively, B outperforms A: MCC is only higher in publication A because the dataset is balanced. The dataset of publication B is imbalanced, with *ϕ* = 0.05. Therefore, MCC is low. Both TPR and TNR, the two metrics that evaluate the classifier independently of the dataset, are higher for classifier B.

Comparing classifiers according to MCC requires that *ϕ* is identical in both datasets. If one applied both classifiers to both datasets, it would become apparent that B outperforms A for either of them (Table 3). Often, reproduction is not possible (because the datasets are not available) or tedious (because retraining the neural networks would consume a lot of time and resources). Therefore, if the goal is to detect the best classifier, we argue against comparing MCC of classifiers from different sources. In such cases, one should rely on BM which is unbiased by class imbalance.

**Table 3:** Evaluation of two classifiers A and B on the same two datasets

Classifier	dataset	TP	FN	TN	FP	*ϕ*	TPR	TNR	BM	MCC		
(a) Relative CM												
A	1	0.35	0.15	0.35	0.15	0.5	0.7	0.7	0.4	0.4		
A	2	0.035	0.015	0.665	0.285	0.05	0.7	0.7	0.4	0.2		
B	1	0.40	0.10	0.40	0.10	0.5	0.8	0.8	0.6	0.6		
B	2	0.04	0.01	0.76	0.19	0.05	0.8	0.8	0.6	0.3		
Classifier	dataset	TP	FN	TN	FP	*ϕ*	TPR	TNR	BM	MCC	n+	n–
(b) Exemplary CM for a sample size of 200												
A	1	70	30	70	30	0.5	0.7	0.7	0.4	0.4	100	100
A	2	7	3	133	57	0.05	0.7	0.7	0.4	0.2	10	190
B	1	80	20	80	20	0.5	0.8	0.8	0.6	0.6	100	100
B	2	8	2	152	38	0.05	0.8	0.8	0.6	0.3	10	190

Ideally, both classifiers are evaluated on both datasets as shown in this table. Otherwise, one should rely on metrics which are independent of the prevalence such as BM. Matthews correlation coefficient (MCC) might be unreliable if one wants to compare classification results across datasets

### Interpretation of high values of multi-category metrics

When performing a binary classification, a practitioner would like to know if all four basic rates (TPR, TNR, PPV and, NPV) are high by checking a single metric. As shown in Eq. 16, MCC generates a high score only if all four of them are high.

BM and BA are calculated based on only TPR and TNR (Eqs. 7 and 6). Therefore, it may seem like BM is unrelated to PPV and NPV, but that is not the case, since these basic rates can be calculated from TPR, TNR and, *ϕ*. Following our redefinition of the confusion matrix (“Redefining confusion matrix in terms of prevalence, TPR and TNR” subsection), we arrive at:

35 $$\begin{array}{*{20}l} {PPV} &= \frac{{TPR} \cdot {\phi}}{{TPR} \cdot {\phi} + (1 - {TNR}) \cdot (1 - {\phi})}  \end{array} $$

36 $$\begin{array}{*{20}l} {NPV} &= \frac{{TNR} \cdot (1 - {\phi})}{(1 - {TPR}) \cdot {\phi} + {TNR} \cdot (1 - {\phi})}  \end{array} $$

**Fig. 3:**
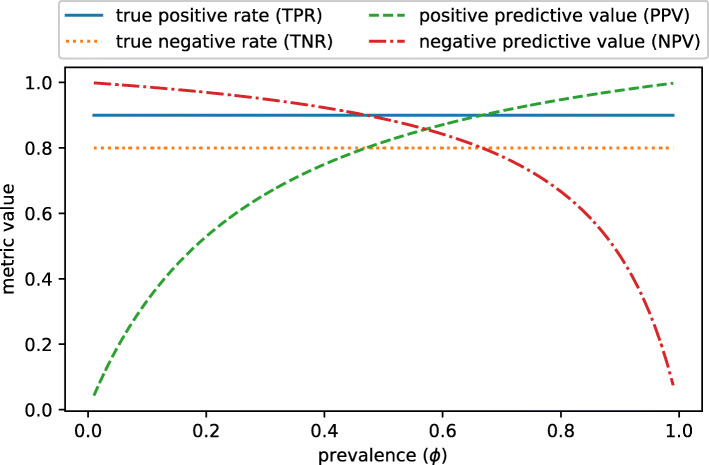
Indicative example with high true positive rate (TPR) and high true negative rate (TNR). We show the trend of the four basic rates if TPR = 0.9 and TNR = 0.8, and illustrate how positive predictive value (PPV) and negative predictive value (NPV) depend on prevalence (*ϕ*). Bookmaker informedness (BM) equals 0.7 in this example. At least one of PPV and NPV is high, even if *ϕ* varies. Only if *ϕ* is close to 0.6, both of them are high

If both TPR and TNR are high, which is the case for big values of BM and BA, at least one of PPV and NPV must be high. One cannot know which one without knowing the prevalence (Fig. 3). Therefore, a high BM guarantees that three of the four basic rates have big values. If the dataset is balanced, both PPV and NPV are high if BM is high.

A high F_1_ score does not guarantee high TNR nor NPV because, for low *ϕ*, PPV can be high even if TNR is low. Accuracy can be rewritten as:

37 $$ accuracy = {TPR} \cdot {\phi} + {TNR} \cdot (1 - {\phi})  $$

Accuracy is high only if: (i) TPR and TNR are high; (ii) TPR and *ϕ* are high; or (iii) TNR is high and *ϕ* is low. In case (i), at least one of PPV and NPV must be high as well. In case (ii), PPV is guaranteed to be high; whereas in case (iii), NPV must be high.

It is well known that accuracy can be large although positive or negative instances are predicted very poorly in highly imbalanced datasets [15].

Similar to Eqs. 35 and 36, TPR and TNR can be expressed in ways of PPV, NPV and, *β*:

38 $$\begin{array}{*{20}l} {TPR} &= \frac{{PPV} \cdot {\beta}}{{PPV} \cdot {\beta} + (1 - {NPV}) \cdot (1 - {\beta})} \end{array} $$

39 $$\begin{array}{*{20}l} {TNR} &= \frac{{NPV} \cdot (1 - {\beta})}{(1 - {PPV}) \cdot {\beta} + {NPV} \cdot (1 - {\beta})} \end{array} $$

If MK is high, at least one of TPR and TNR must therefore be high as well. Similar to the discussion above for BM, we cannot know which one without having identified *β*.

We can summarize that a high F_1_ score or accuracy guarantee that two of the basic rates are high (Table 4). High BA, BM or MK guarantee that three of the basic rates are high. A high MCC is the only multi-category metric discussed in this study that guarantees that all four basic rates are high.

**Table 4:** Recap of the relationship between the multi-category metrics and the basic rates of the confusion matrix

Scenario	Basic rates condition	# guaranteed high basic rates
high MCC means:	high TPR, TNR, PPV, and NPV	4
high BA means:	high TPR, TNR, and at least one of PPV and NPV	3
high BM means:	high TPR, TNR, and at least one of PPV and NPV	3
high MK means:	high PPV, NPV, and at least one of TPR and TNR	3
high F_1_ score means:	high PPV and TPR	2
high accuracy means:	high TPR and PPV, or high TNR and NPV	2

#: integer number. MCC: Matthews correlation coefficient (Eq. 5). BA: balanced accuracy (Eq. 6). BM: bookmaker informedness (Eq. 7). MK: markedness (Eq. 8). F_1_ score: harmonic mean of TPR and PPV (Supplementary information). Accuracy: ratio between correctly predicted data instances and all data instances (Supplementary information). We call “basic rates” these four indicators: TPR, TNR, PPV, and NPV

### Bookmaker informedness is the only metric that measures how similar a classifier is to random guessing

Often, papers introduce MCC with the notion that MCC = 0 signals that classifier performance is no better than random guessing whereas a perfect classifier would have MCC = +1. Although these statements are correct, one should avoid the misconception that MCC is a robust indicator of how (dis)similar a classifier is to random guessing. Only BM, and BA, can address this topic truthfully without any distortion by *ϕ* and *β*.

Consider the example of three students taking an exam consisting of yes/no questions, a scenario which is analogous to binary classification. The students randomly guess “yes” and “no” to fill in the exam sheet because they did not prepare. The professor has the correct solutions on their desk. While the professor is distracted, the students take the opportunity to cheat and look up a fraction of the answers. The first one copies 25% of the correct answers, the second one 50%, and the third one 75%.

**Fig. 4:**
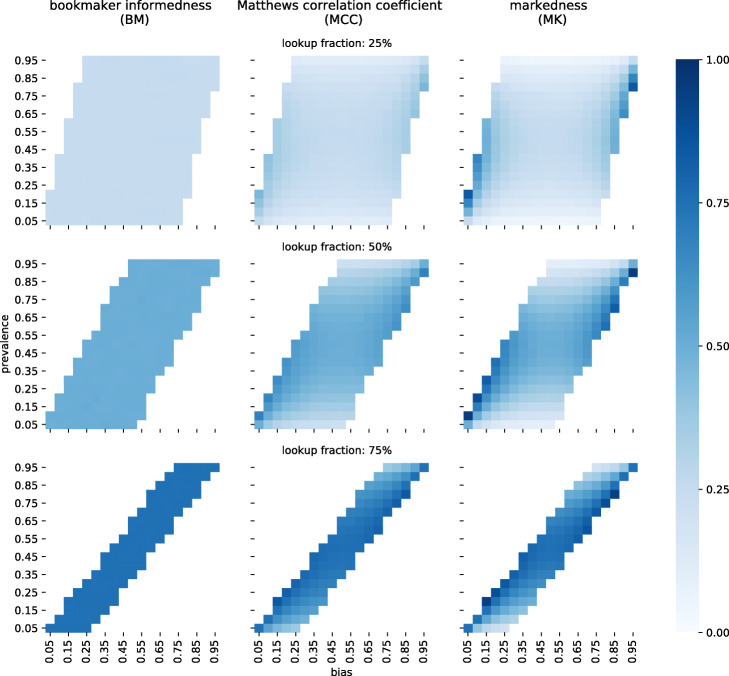
BM, MCC and MK for classifiers with known randomness. We simulated classifiers with known amounts of randomness. To that purpose, we generated random lists of reference classes with a given prevalence. A fraction of those classes were copied (called lookup fraction) and used as predicted labels, the remaining ones were generated randomly, matching a given bias. Matching the reference classes with the prediction labels we determined bookmaker informedness, Matthews correlation coefficient and markedness (left, center and right column respectively). The rows differ by the amount of randomness/lookup fraction

We calculate BM, MCC and MK for the exams of the three students and display the results in Fig. 4. BM is not at all affected by *ϕ* or *β* and always equals the lookup fraction. *ϕ* corresponds to the share of questions where the correct answer would be “yes” whereas *β* is the share of questions the students answered with "yes". In fact, we can define the randomness of a classifier to be equal to 1−∥*BM*∥. This will provide the correct answer to the question: “How likely is the classifier to guess at random?”.

MCC and even more so MK can deviate from the respective lookup fraction if *ϕ* and *β* are dissimilar. In this example, we know exactly that the students are 25%, 50% or 75% informed since they have to randomly guess the rest. This fact is independent of *ϕ* and *β*. Therefore, neither MCC nor MK yield a reliable estimate of how similar to random guessing the answers of students are.

This extends to other classifiers. A value of MCC or MK close to zero should not be considered evidence that a classifier is similar to random guessing. We note that this deviation becomes even more extreme if *ϕ* or *β* approaches zero or one.

### Ranking of classifiers in a real bioinformatics scenario

Similar to what we did for the study where we compared MCC with accuracy and F_1_ score [15], here we show a real bioinformatics scenario where the Matthews correlation coefficient result being more informative than the other rates.

**Dataset**. We applied several supervised machine learning classifiers to microarray gene expression of colon tissue collected by Alon and colleagues [57], who released it publicly within the Partial Least Squares Analyses for Genomics (plsgenomics) R package [58, 59]. This dataset comprises 2,000 gene probesets of 62 subjects; 22 of these subjects are healthy controls and 40 have colon cancer (that is 35.48% negatives and 64.52% positives) [60].

**Experiment design**. We used four machine learning binary classifiers to predict patients and healthy controls in this dataset: Decision Tree [61], *k*-Nearest Neighbors (*k*-NN) [62], Naïve Bayes [63], and Support Vector Machine with radial Gaussian kernel [64].

Regarding Decision Trees and Naïve Bayes, we trained the classifiers on a training set containing 80% of randomly selected data instances, and tested them on a test set consisting of the remaining 20% data instances. For *k*-Nearest Neighbors (*k*-NN) and SVM, instead, we divided the dataset into training set (60% data instances, randomly selected), validation set (20% data instances, randomly selected), and the test set (remaining 20% data instances). We took advantage of the validation set for the hyper-parameter optimization grid search [16]: number *k* of neighbors for *k*-NN, and cost *C* value for the SVM. For all the classifiers, we repeated the execution 10 times and registered the average score for MCC, balanced accuracy, bookmaker informedness, markedness, and the four basic rates (true positive rate, true negative rate, positive predictive value, and negative predictive value).

We then ranked the results achieved on the test sets or the validation sets first based on MCC, then based on BA, then on BM, and finally based on MK (Table 5). For the sake of simplicity, we do not consider the uncertainty in the metrics caused by the limited sample size and the resulting uncertainty in the rankings [53].

**Table 5:** Bioinformatics scenario: binary classification of colon tissue gene expression

Rank	Method	MCC	BA	BM	MK	TPR	TNR	PPV	NPV
	**MCC ranking:**								
1	Decision Tree	**0.447**	0.715	0.429	0.477	0.728	0.701	0.774	0.702
2	Radial SVM	**0.423**	0.695	0.390	0.517	0.891	0.498	0.754	0.726
3	*k*-Nearest Neighbors	**0.418**	0.706	0.412	0.443	0.887	0.525	0.826	0.617
4	Naïve Bayes	**0.408**	0.722	0.444	0.375	0.778	0.667	0.875	0.500
	**BA ranking:**								
1	Naïve Bayes	0.408	**0.722**	0.444	0.375	0.778	0.667	0.875	0.500
2	Decision Tree	0.447	**0.715**	0.429	0.477	0.728	0.701	0.774	0.702
3	*k*-Nearest Neighbors	0.418	**0.706**	0.412	0.443	0.887	0.525	0.826	0.617
4	Radial SVM	0.423	**0.695**	0.390	0.517	0.891	0.498	0.754	0.726
	**BM ranking:**								
1	Naïve Bayes	0.408	0.722	**0.444**	0.375	0.778	0.667	0.875	0.500
2	Decision Tree	0.447	0.715	**0.429**	0.477	0.728	0.701	0.774	0.702
3	*k*-Nearest Neighbors	0.418	0.706	**0.412**	0.443	0.887	0.525	0.826	0.617
4	Radial SVM	0.423	0.695	**0.390**	0.517	0.891	0.498	0.754	0.726
	**MK ranking:**								
1	Radial SVM	0.423	0.695	0.390	**0.517**	0.891	0.498	0.754	0.726
2	Decision Tree	0.447	0.715	0.429	**0.477**	0.728	0.701	0.774	0.702
3	*k*-Nearest Neighbors	0.418	0.706	0.412	**0.443**	0.887	0.525	0.826	0.617
4	Naïve Bayes	0.408	0.722	0.444	**0.375**	0.778	0.667	0.875	0.500

Radial SVM: Support Vector Machine with radial Gaussian kernel. Positives: patients diagnosed with colon cancer. Negatives: healthy controls. MCC: Matthews correlation coefficient(Eq. 5). BA: balanced accuracy (Eq. 6). BM: bookmaker informedness (Eq. 7). MK: markedness (Eq. 8). TPR: true positive rate. TNR: true negative rate. PPV: positive predictive value. NPV: negative predictive value. MCC, BM, MK value interval: [−1,+1]. BA, TPR, TNR, PPV, NPV value interval: [0,1].

Bold values represent the corresponding ranking for each metric

**Results: different metric, different ranking**. The four rankings we employed to report the same results show two interesting aspects. First, the top classifier changes when we the ranking rate changes.

In the MCC ranking, in fact, the top performing method is Decision Tree (MCC = +0.447), while in the balanced accuracy ranking and in the bookmaker informedness ranking the best classifier resulted being radial Naïve Bayes (BA = 0.722 and BM = 0.444). And in the markedness ranking, even a different classifier occupied the top position: Radial SVM with MK = 0.575. The ranks of the methods change, too: Decision Tree is ranked first in the MCC ranking, but ranked second in the other rankings. And radial SVM changes its rank in 3 rankings out of 4, occupying the second position in the MCC standing, last position in the BA and BM standings, and top position in the MK standing. Only the rankings of balanced accuracy and bookmaker informedness have the same standing, and that comes with no surprise since they contain equivalent information, as we mentioned earlier (“BA and BM contain equivalent information” subsection).

A machine learning practitioner, at this point, could ask the question: which ranking should I choose? As we explained earlier, in case the practitioner wants to give the same importance to negatives and positives as well as to informedness and markedness, we suggest to focus on the ranking obtained by the MCC. A high value of MCC, in fact, would mean that the classifier was able to correctly predict the majority of the positive data instances (TPR) and the majority of the negative data instances (TNR), and to correctly make the majority of positive predictions (PPV) and the majority of negative predictions (NPV). So, if the practitioner decided to base her/his study on the results of the MCC, she/he would have more chances to have high values for the four basic rates, than by choosing BA, BM, or MK.

Finally, we note that the differences in MCC between classifiers are small, whereas differences of the four basic rates are relatively large. If it was desirable to have all basic rates at a similar level, also meaning that none of them are low, Decision Tree would be the best choice of classifiers in this scenario. SVM and *k*-Nearest Neighbors have high sensitivity at the expense of specificity. Instead, if a high precision here was needed and a low NPV was acceptable, the Naive Bayes classifier would be the most promising choice in this setting. MCC does not capture these details: it only measures how well the classifiers perform on all four basic rates together. Although MCC states that the classifiers perform similarly well, we can see that they have both advantages and disadvantages, if compared to each other in more detail.

## Conclusions

The evaluation of binary classifications is an important step in machine learning and statistics, and the four-category confusion matrix has emerged as one of the most powerful and efficient tools to perform it. To recap the meaning of a two-class confusion matrix, researchers have introduced several statistical metrics, such as Matthews correlation coefficient (MCC), accuracy, balanced accuracy (BA), bookmaker informedness (BM), markedness (MK), F_1_ score, and others. Since the advantages of Matthews correlation coefficient over accuracy and F_1_ score have been already unveiled in the past [15], in this study we decided to compare MCC with balanced accuracy, bookmaker informedness, and markedness, by exploring their mathematical relationships and by analyzing some use cases.

From our analysis, we can confirm again that MCC results are generally more informative and truthful than BA, BM, and MK if the positive class and the negative class of the dataset have the same importance in the analysis, and if correctly classifying the existing ground truth data instances has the same importance of making correct predictions in the analysis. Additionally, we can state that a high Matthews correlation coefficient (close to +1) means always high values for all the four basic rates of the confusion matrix: true positive rate (TPR), true negative rate (TNR), positive predictive value (PPV), and negative predictive value (NPV) (Table 4). The same deduction cannot be made for balanced accuracy, bookmaker informedness, and markedness.

The situation changes if correctly classifying the existing ground truth data instances is more important than correctly making predictions: in this case, BA and BM can be more useful than MCC. Similarly, if making correct predictions is more relevant than correctly identifying ground truth data instances, MK can be more informative than MCC. Instead, if the positive data instances are more important than negative elements in a classification (both for ground truth classification and for predictions), F_1_ score can be more relevant than Matthews correlation coefficient.

In our analysis, we also showed two specific tasks for which bookmaker informedness can be more useful than the other confusion matrix rates: to make a fair comparison between two different classifiers evaluated on different datasets, and to detect the similarity of a classification to random guessing. Moreover, we reported a real bioinformatics scenario, where the usage of different rates can influence the ranking of classifiers applied to microarray gene expression.

To conclude, as a general rule of thumb, we suggest the readership to focus on MCC over BA, BM, and MK for any study by default, and to try to obtain a value close to +1 for it: achieving MCC = +0.9, for example, guarantees very high F_1_ score, accuracy, markedness, balanced accuracy, and bookmaker informedness, and even very high sensitivity, specificity, precision, and negative predictive value. If a specific class is considered more important (for example, predictions over ground truth classifications, or positives over negatives), or the goal of the study is the comparison of classifiers across datasets or the evaluation of the level of random guessing, we advise the practitioner to shift to BA, BM, MK, or F_1_ score, as mentioned earlier.

In the future, we plan to compare the Matthews correlation coefficient with other metrics, such as Brier score [65], Cohen’s Kappa [66], K measure [67], Fowlkes-Mallows index [68] and H-index [69].

## Supplementary Information

**Additional file 1** Known randomness simulation algorithm and formulas of the additional metrics.

## Data Availability

Our software code is publicly available under the GNU General Public License version 3 (GPL 3.0) at: https://github.com/niklastoe/MCC_BM_BA_MK

## Abbreviations

AUC: Area under the curve
BA: Balanced accuracy
BM: Bookmaker informedness
MCC: Matthews correlation coefficient
MK: Markedness
NPV: Negative predictive value
PPV: Positive predictive value
PR: Precision-recall
ROC: Receiver operating characteristic
TNR: True negative rate
TPR: True positive rate
