# Supplementary material for: The Matthews correlation coefficient (MCC) is more reliable than balanced accuracy, bookmaker informedness, and markedness in two-class confusion matrix evaluation
Source: BioData Min. 2021 Feb 4;14:13. doi: 10.1186/s13040-021-00244-z (PMC7863449; doi:10.1186/s13040-021-00244-z)
Supplement: Supplementary file 1 — Additional file 1 Known randomness simulation algorithm and formulas of the additional metrics. [file 13040_2021_244_MOESM1_ESM.pdf]

Supplementary information

Known randomness simulation algorithm

In this section, we report the pseudo-code description of the known randomness simulation algorithm we used (algorithm 1). The implementation in `Python` can be found online ("Availability of data and software" section).

Algorithm 1: Simulate classifier with known randomness

```
# generate reference classes and predictions randomly;
N = 500,000;
reference = Bernoulli(p=φ, sample_size=N);
;
# look up a percentage of the reference data;
lookup = reference[:N*lookup_fraction];
# generate random predictions so the overall bias is correct;
remaining_bias = (β - lookup_fraction * φ) / ( 1 - lookup_fraction);
random_prediction = Bernoulli(p=remaining_bias, sample_size=N * (1-lookup_fraction));
# combine lookup with random predictions and consider these the predicted labels;
predicted_labels = concatenate([lookup, random_prediction]);
;
# calculate metrics;
bm_val = bm(reference, prediction);
mcc_val = mcc(reference, prediction);
mk_val = mk(reference, prediction)
```

Additional metrics

In this section, we report the formulas of the additional metrics we employed in the manuscript.

$$\text{false detection rate (FDR)} = 1 - PPV = \frac{FP}{TP + FP} \tag{40}$$

$$(\text{worst value} = 1; \text{best value} = 0)$$

$$\text{false omission rate (FOR)} = 1 - NPV = \frac{FN}{TN + FN} \tag{41}$$

$$(\text{worst value} = 1; \text{best value} = 0)$$

$$\text{false positive rate (FPR)} = 1 - TNR = \frac{FP}{TN + FP} \tag{42}$$

$$(\text{worst value} = 1; \text{best value} = 0)$$

$$\text{false negative rate (FNR)} = 1 - TPR = \frac{FN}{TP + FN} \tag{43}$$

$$(\text{worst value} = 1; \text{best value} = 0)$$

$$\mathrm{F}_1 \text{ score} = \frac{2 \cdot TP}{2 \cdot TP + FP + FN} = 2 \cdot \frac{PPV \cdot TPR}{PPV + TPR} \tag{44}$$

$$(\text{worst value} = 0;\ \text{best value} = 1)$$

$$\text{accuracy} = \frac{TP + TN}{TP + FN + TN + FP} \tag{45}$$

$$(\text{worst value} = 0;\ \text{best value} = 1)$$

Relationship between rates expressed through Pearson correlation coefficient

n	PCC(MCC, BM)	PCC(MCC, MK)	PCC(BM, MK)
5	0.99385	0.99385	0.97566
6	0.99120	0.99120	0.96534
7	0.98905	0.98905	0.95716
8	0.98736	0.98736	0.95080
9	0.98602	0.98602	0.94588
10	0.98497	0.98497	0.94210
10	0.98497	0.98497	0.94210
11	0.98415	0.98415	0.93920
12	0.98351	0.98351	0.93699
13	0.98301	0.98301	0.93532
14	0.98262	0.98262	0.93406
15	0.98232	0.98232	0.93314
16	0.98210	0.98210	0.93247
17	0.98193	0.98193	0.93201
18	0.98181	0.98181	0.93172
19	0.98173	0.98173	0.93156
20	0.98168	0.98168	0.93150
21	0.98166	0.98166	0.93153
22	0.98165	0.98165	0.93162
23	0.98166	0.98166	0.93176
24	0.98169	0.98169	0.93195
25	0.98173	0.98173	0.93217
50	0.98344	0.98344	0.93926
75	0.98470	0.98470	0.94389
100	0.98550	0.98550	0.94670
200	0.98691	0.98691	0.95152
300	0.98743	0.98743	0.95326
400	0.98770	0.98770	0.95415
500	0.98786	0.98786	0.95469
1,000	0.98819	0.98819	0.95577
10,000	0.99345	0.99345	0.97527
100,000	0.99346	0.99346	0.97527
1,000,000	0.99346	0.99346	0.97527
1,000,000,000	0.99346	0.99346	0.97527

Table S1: ***Pearson correlation between MCC, BM and MK.*** *Metrics values are computed on all confusion matrices with given number of samples for* $N \leq 1000$*, and on a subset of* 10^8 *confusion matrices for* $N \geq 10^4$ *(in italic).*
